# Association between smartphone ownership, proficiency, and electronic money use among community-dwelling older adults: a cross-sectional study

**DOI:** 10.3389/fdgth.2026.1947033

**Published:** 2026-10-20

**Authors:** Suguru Shimokihara, Takayuki Tabira, Michio Maruta, Hyuma Makizako, Gwanghee Han, Taishiro Kamasaki, Yoshihiko Akasaki, Yuma Hidaka, Yusuke Kumura, Wataru Kukizaki, Mitsuki Kojima, Takuro Kubozono, Mitsuru Ohishi

**Affiliations:** 1 Graduate School of Biomedical Sciences, Health Sciences, Nagasaki University, Nagasaki, Japan; 2 Visiting Researcher, Faculty of Medicine, Kagoshima University, Kagoshima, Japan; 3 Department of Occupational Therapy, School of Health Sciences, Sapporo Medical University, Sapporo, Japan; 4 Department of Occupational Therapy, School of Health Sciences, Faculty of Medicine, Kagoshima University, Kagoshima, Japan; 5 Department of Physical Therapy, School of Health Sciences, Faculty of Medicine, Kagoshima University, Kagoshima, Japan; 6 Department of Occupational Therapy, School of Health Sciences at Fukuoka, International University of Health and Welfare, Fukuoka, Japan; 7 Faculty of Rehabilitation Sciences Department of Rehabilitation Sciences, Nishikyushu University, Kanzaki, Saga, Japan; 8 Graduate School of Health Sciences, Kagoshima University, Kagoshima, Japan; 9 Division of Occupational Therapy, Department of Rehabilitation, Faculty of Health Science, Kumamoto Health Science University, Kumamoto, Japan; 10 Department of Cardiovascular Medicine and Hypertension, Graduate School of Medical and Dental Sciences, Kagoshima University, Kagoshima, Japan

**Keywords:** aging in place, digital financial management, electronic money, instrumental activities of daily living, older adults, smartphone proficiency

## Abstract

**Background:**

Electronic money (e-money) is increasingly used for daily activities, including shopping and transportation. As cashless payments become more common, older adults may need digital devices to manage everyday financial transactions. However, the relationship between smartphone ownership, proficiency, and e-money use remains unclear. This study examined these associations among community-dwelling older adults.

**Methods:**

This cross-sectional study analyzed data from a community-based health check-up program involving 474 community-dwelling adults aged ≥65 years. E-money use was assessed using the “Use electronic money” subitem of the managing finances domain in the Process Analysis of Daily Activity for Dementia. Participants were classified as e-money users if they performed all three actions: presenting an e-money card or device to a reader, adding funds, and understanding the remaining balance. Smartphone ownership was assessed by questionnaire, and proficiency among owners was measured using the Japanese short version of the Mobile Device Proficiency Questionnaire. Participants were classified as smartphone nonowners (*n* = 62) or owners with low (*n* = 138), moderate (*n* = 137), or high (*n* = 137) proficiency. Modified Poisson regression with robust error variance estimated prevalence ratios (PRs) and 95% confidence intervals (CIs).

**Results:**

Of 474 participants, 200 were e-money users and 274 were nonusers. E-money users were younger, had more years of education, and more frequently had high smartphone proficiency than nonusers. After adjustment, moderate (PR 2.54, 95% CI 1.31–4.93) and high (PR 3.65, 95% CI 1.89–7.06) smartphone proficiency were significantly associated with a higher prevalence of e-money use compared with smartphone nonownership. Similar associations were observed in analyses restricted to smartphone owners.

**Conclusions:**

Moderate and high smartphone proficiency, but not low proficiency, were associated with a higher prevalence of e-money use compared with smartphone nonownership. Similar findings among smartphone owners suggest that e-money use may represent a contemporary financial instrumental activities of daily living requiring practical digital device proficiency.

## Introduction

Supporting older adults to age in place is an important public health and social policy priority in rapidly aging societies. From a healthy aging perspective, maintaining functional ability enables older adults to live independently, participate in society, and continue valued activities (1). Instrumental activities of daily living (IADL) are particularly important for independent community living because they require complex cognitive, physical, and social abilities (2). Identifying factors associated with maintaining IADL is therefore central to strategies supporting aging in place. Among IADL domains, financial management is particularly cognitively demanding and vulnerable to cognitive decline. Financial capacity may decline in the early stages of cognitive impairment, including mild cognitive impairment, and difficulties in money management may precede more apparent functional dependence (3–5) Thus, financial management is both an indicator of functional independence and an essential domain for continued independent living in the community.

However, contemporary financial management extends beyond conventional cash handling, bankbook management, and face-to-face transactions. The rapid expansion of cashless payment systems has transformed everyday financial activities, with smartphones playing an important role in this transition. Smartphones are increasingly common among older adults and provide access to communication, information, health management, transportation, and financial services. Many digital payment services and electronic money (e-money) are integrated into these device-based activities. E-money is defined as monetary value stored electronically on a technical device, such as a card or computer, that can be used to make payments to entities other than the issuer (6). In Japan, prepaid e-money is generally regulated as a prepaid payment instrument under the Payment Services Act, covering prepaid monetary value recorded electronically or magnetically and used to purchase goods or services. The Japanese payment environment is characterized by widespread use of rechargeable transportation IC cards, such as Suica and PASMO. Since nationwide interoperability among major transportation IC cards was introduced in 2013, these cards have become widely usable across railway and bus networks and as e-money at retail stores, vending machines, and other everyday settings. Thus, e-money use in Japan includes both card-based and device-based payments and is not synonymous with smartphone-based mobile payment. Although credit cards and prepaid e-money are formally distinct payment instruments, users may perceive them less distinctly in everyday cashless transactions. As payment functions are increasingly integrated into cards, smartphones, and applications, these services may represent a broader continuum of electronic cashless payments. Reflecting this diffusion, Japan's cashless payment ratio reached 58.0% in 2025, with electronic money accounting for approximately ¥6.0 trillion in payment value (7). E-money and related digital payment services are therefore increasingly integrated into daily activities, including shopping, transportation, and social participation.

This transformation suggests that financial management in later life may be considered a digitally mediated IADL requiring not only numeracy and memory but also the ability to operate digital devices. Prior research has emphasized that smartphone ownership alone is insufficient to explain how older adults use digital technologies in everyday life (8). Mobile device proficiency, defined as the ability to use smartphones and tablets to perform various tasks, may be a more meaningful indicator of digital capability among older adults (9). Among community-dwelling older adults, higher smartphone proficiency has been associated with higher-level competence and physical function, suggesting that it may reflect broader life competence in the digital era (10). However, our previous work did not examine whether smartphone proficiency is associated with performing a specific digitally mediated daily activity, particularly financial activities.

Digital financial literacy provides a useful framework for examining this issue. It encompasses conventional financial knowledge and skills as well as competencies required to use digitally delivered financial products and services, operate digital tools, and recognize and manage risks associated with digital transactions (11–13) From this perspective, smartphone access may represent one component of participation in digital financial activities, whereas practical proficiency in using digital devices may also be relevant to whether such activities can be performed in everyday life. Studies of mobile payment adoption among older adults have primarily examined behavioral intention and psychosocial determinants, including perceived usefulness, ease of use, attitude, trust, and perceived risk (14, 15). Less is therefore known about the relationship between smartphone proficiency and actual e-money use in everyday life. The present study extends this literature in three respects. First, it examines actual e-money use rather than intention to adopt mobile payment services. Second, e-money use was evaluated through a face-to-face, assessor-administered assessment of participants' everyday financial activities. Third, the assessment included both card-based and device-based e-money rather than only smartphone-based payment services.

Accordingly, this study aimed to examine the associations of smartphone ownership and proficiency with e-money use among community-dwelling older adults. Based on the digital financial literacy framework, we hypothesized that e-money use would differ by smartphone proficiency level, with moderate- and high-proficiency groups showing greater use than groups with limited access or proficiency, even after accounting for relevant sociodemographic, cognitive, health-related, and financial management factors. Clarifying these associations may help identify older adults requiring additional support to participate in increasingly cashless environments and inform educational and support strategies for digital financial activities in later life.

## Method

### Participants

This cross-sectional study used data from a community-based health check-up program conducted in 2025 in Tarumizu City, Kagoshima Prefecture, Japan. The program targeted community residents aged ≥40 years, who were invited through pre-distributed response postcards, local publicity campaigns, and newspaper advertisements. The present analysis focused on older adults aged ≥65 years. Participants were excluded if they were aged <65 years (*n* = 273), had a self-reported history of dementia (*n* = 3), required assistance with daily self-care (*n* = 44), were certified as needing care under the Japanese Long-Term Care Insurance system (*n* = 2), or had missing data for key study variables (*n* = 5). Figure 1 shows the participant selection process. This cross-sectional study was reported according to the STROBE guidelines, and the completed checklist is provided in Supplementary Table S1.

**Figure 1:**
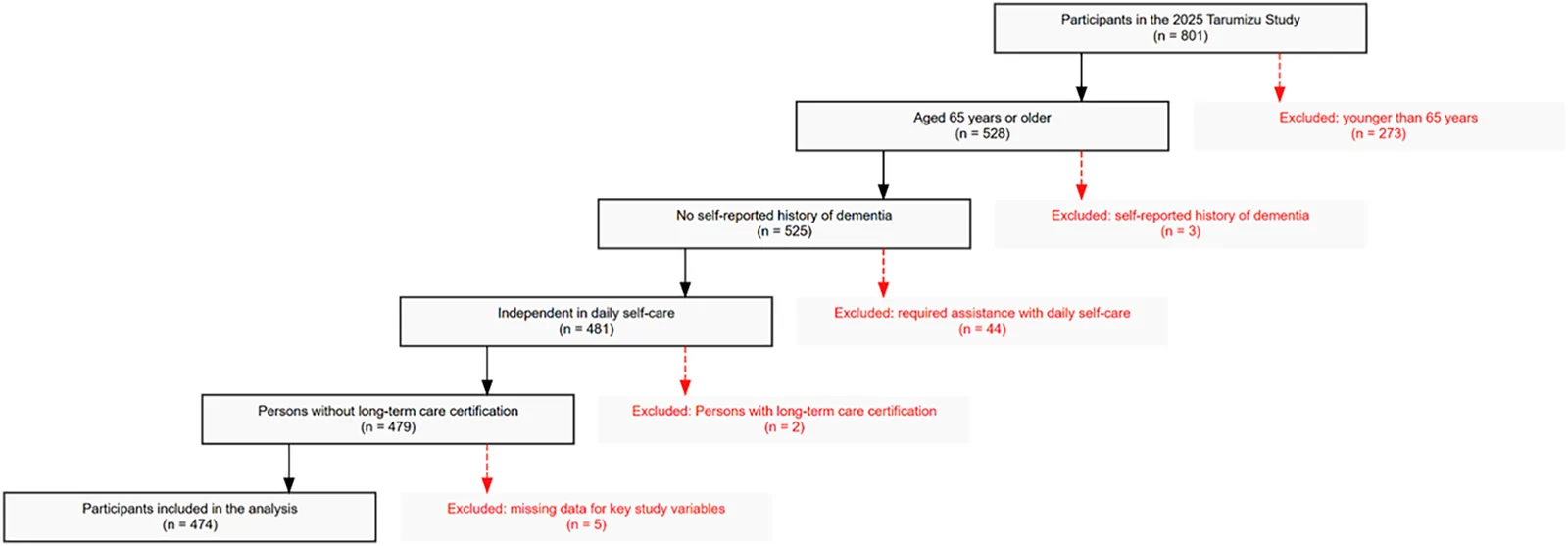
Study flowchart.

### Ethical considerations

The study protocol was approved by the Ethics Committee of the Faculty of Medicine, Kagoshima University (#170351-R13). Written informed consent was obtained from all participants before enrollment. All study procedures were conducted in accordance with the principles of the Declaration of Helsinki.

### Assessment of e-money use

In this study, e-money was operationally defined as electronic cashless payment methods used in daily life. The assessment broadly captured payment methods commonly used in Japan, including transportation IC cards such as Suica and PASMO, credit cards, and device-based or QR-code payment services such as PayPay. Thus, “e-money” was used as a practical umbrella term for electronic cashless payments rather than being restricted to prepaid stored-value instruments. The assessment captured card-based and device-based payment behaviors and was not limited to smartphone-based payment applications. E-money use was assessed using the Process Analysis of Daily Activity for Dementia (PADA-D) (16). The PADA-D is an activities of daily living assessment tool originally developed for people with dementia. It evaluates daily activities by dividing each activity into sequential processes and specific constituent actions. It comprises six basic activities of daily living (BADL) domains (feeding, bathing, dressing, grooming, mobility, and toileting) and eight IADL domains (cooking, housework, shopping, ability to use the telephone, use of transportation, laundry, managing medication, and managing finances). Each activity comprises five sequential processes, each consisting of three specific actions. Each action is scored as 1 if performed and 0 if not performed. The PADA-D has demonstrated high internal consistency and criterion validity (16). In the present study, trained assessors evaluated e-money use through a face-to-face, one-on-one assessment using the “Use electronic money” subitem within the managing finances domain of the PADA-D. This subitem comprises the following three actions: holding an electronic money card or device over a reader, adding funds to the e-money balance, and understanding the remaining balance. Participants were classified as e-money users only if they reported performing all three actions.

### Assessment of smartphone ownership and proficiency

Smartphone ownership was assessed using a self-administered questionnaire. Participants reported whether they currently owned a smartphone and were classified as smartphone owners or nonowners. Smartphone proficiency was assessed only among smartphone owners using the Japanese version of the Mobile Device Proficiency Questionnaire short version (MDPQ-16) (17). The MDPQ-16 assesses proficiency with mobile devices, including smartphones and tablets, across eight domains: mobile device basics, communication, data and file storage, internet, calendar, entertainment, privacy, and troubleshooting and software management. Thus, MDPQ-16 scores indicated mobile device proficiency among smartphone owners. Among smartphone nonowners, four participants reported owning a tablet computer; they remained in the smartphone nonownership group because the exposure was defined specifically by smartphone ownership. Participants rated their ability to perform 16 mobile-device-related tasks on a 5-point scale: 1 = never tried, 2 = not at all, 3 = not very easy, 4 = somewhat easy, and 5 = very easy. For each domain, the mean of the two item scores is calculated, and the eight domain means are summed to obtain the total MDPQ-16 score. Total scores range from 8 to 40, with higher scores indicating greater mobile device proficiency. The original MDPQ-16 has demonstrated good internal consistency (17), and a previous systematic review suggested that it is an appropriate measure of digital literacy among older adults because it comprehensively assesses mobile device proficiency (18). For the present analysis, participants were categorized into four groups according to smartphone ownership and proficiency. Participants without a smartphone were classified as nonowners. Among smartphone owners, MDPQ-16 scores were divided into tertiles and classified as low (8.0–16.5 points), moderate (17.0–25.5 points), or high (26.0–40.0 points) smartphone proficiency. Thus, the final variable comprised four categories: nonownership, low proficiency, moderate proficiency, and high proficiency.

### Covariates

#### Social factors

Social factors included age, sex, years of education, living alone, marital status, employment status, and driving status. Age and years of education were treated as continuous variables. Sex was self-reported using a questionnaire with male and female response categories. Gender identity was not assessed. Living arrangement was categorized as living alone or living with someone.

Marital status was assessed by asking participants, “Are you currently married?” Response options were never married, currently married or living with a partner, widowed, and divorced. For analysis, marital status was categorized into three groups: married, widowed or divorced, and never married.

Employment status was assessed by asking participants, “Are you currently engaged in paid work?” Participants responded yes or no.

Driving status was assessed based on participants' current driver's license status and driving behavior. Response options were never had a driver's license, previously had a driver's license but had returned it, had a driver's license but had not renewed it, had a valid driver's license but did not currently drive, and had a valid driver's license and currently drove. Participants with a valid driver's license who currently drove were classified as having a current driving habit. Current driving habit was included as a covariate because driving status is closely related to community mobility and engagement in IADLs among older adults, including shopping and other out-of-home activities (19). Because these activities provide opportunities to encounter and use e-money, driving habit was considered a potential confounder reflecting differences in community mobility and exposure to payment contexts.

#### Functional factors

Functional factors included physical function, cognitive function, and depressive symptoms.

Physical function was assessed using grip strength and usual gait speed. Physical impairment was defined as weakness or slowness. Weakness was defined as grip strength <28 kg for males and <18 kg for females, and slowness as usual gait speed <1.0 m/s. Participants meeting either criterion were classified as having physical impairment. These cutoff values are widely used and have well-established consensus for screening physical frailty and sarcopenia among Asian older adults (20, 21).

Cognitive function was assessed using the National Center for Geriatrics and Gerontology–Functional Assessment Tool (NCGG-FAT) (22). The NCGG-FAT evaluates four cognitive domains: memory, attention, executive function, and processing speed. Memory was assessed using word list tasks involving immediate recognition and delayed recall. Attention and executive function were assessed using tablet versions of the Trail Making Test Parts A and B, respectively. Processing speed was assessed using the tablet version of the Symbol Digit Substitution Task. The NCGG-FAT has demonstrated moderate to high validity and high test-retest reliability among community-dwelling older adults (22, 23). Lower cognitive status was defined as a score <1.5 SD of the age- and education-specific mean in at least one cognitive domain (24).

Depressive symptoms were assessed using the 15-item Geriatric Depression Scale (GDS-15), a widely used screening measure for depressive symptoms in older adults (25–27) Total scores range from 0 to 15, with higher scores indicating more severe depressive symptoms. The GDS-15 score was treated as a continuous variable in the analyses.

#### General financial management ability

General financial management ability was assessed using the managing finances domain of the PADA-D, excluding the “Use electronic money” item. This variable was included to distinguish e-money use from conventional financial management ability. The assessment comprised four items and 12 actions: handling cash, using cash in daily life, understanding household finances, and using banks or post offices. Participants who performed all 12 actions were classified as independent in general financial management; those who did not perform at least one action were classified as not independent.

### Statistical analyses

Participant characteristics were summarized according to e-money use, with participants classified as e-money users or nonusers. Continuous variables are presented as medians and interquartile ranges (IQRs), and categorical variables as numbers and percentages. Between-group differences were examined using the Wilcoxon rank sum test for continuous variables and the chi-square test or Fisher exact test for categorical variables, depending on expected cell counts.

Modified Poisson regression with robust error variance was used to examine the association between smartphone ownership/proficiency and e-money use. E-money use was the dependent variable, and smartphone ownership/proficiency was the main independent variable, categorized as smartphone nonownership, low smartphone proficiency, moderate smartphone proficiency, or high smartphone proficiency. Smartphone nonownership was the reference category. Modified Poisson regression was used because e-money use was binary and expected to be relatively common. In such situations, odds ratios from logistic regression may overestimate the association and be less intuitive to interpret. Modified Poisson regression with robust error variance directly estimates prevalence ratios (PRs), which are more interpretable for cross-sectional studies with binary outcomes (28, 29). Three regression models were constructed. Model 1 was adjusted for social factors: age, sex, years of education, living alone, marital status, employment status, and current driving habit. Model 2 was additionally adjusted for functional factors: physical impairment, cognitive impairment, and depressive symptoms. Model 3 was further adjusted for general financial management ability to account for the possibility that e-money use reflects broader financial management capacity rather than smartphone-related proficiency alone.

To distinguish associations with smartphone proficiency from those with smartphone access, additional analyses were restricted to 412 smartphone owners, with the low-proficiency group as the reference category. Smartphone proficiency was categorized as low, moderate, or high, and the same covariate adjustment strategy as in the main analysis was applied.

As a sensitivity analysis, smartphone proficiency was examined as a continuous variable among smartphone owners to determine whether the association with e-money use depended on sample-specific tertile cut points. The MDPQ-16 score was standardized, and PRs per 1 SD increase were estimated using modified Poisson regression. The same covariate adjustment strategy as in the categorical analysis was applied.

To evaluate the potential influence of unmeasured confounding, E-values were calculated for PRs from the adjusted models. E-values were calculated for both the main analysis, which used smartphone nonownership as the reference category, and the analysis restricted to smartphone owners, which used low smartphone proficiency as the reference category. The E-value represents the minimum strength of association an unmeasured confounder would need with both the exposure and outcome, after accounting for measured covariates, to explain away the observed association (30, 31). E-values were calculated for the point estimates and lower limits of the 95% confidence intervals (CIs).

Results are presented as PRs with 95% CIs. All statistical tests were two-sided, and *P* < .05 was considered statistically significant. All analyses were performed using R version 4.4.2 (R Foundation for Statistical Computing, Vienna, Austria). Data management and descriptive analyses were conducted primarily using the dplyr package (ver. 1.2.0). Modified Poisson regression models were fitted using the glm function in the stats package, with robust variance estimates obtained using the sandwich (ver. 3.1.1) and lmtest (ver. 0.9.40) packages.

## Results

### Participant characteristics

A total of 474 community-dwelling older adults were included in the analysis. Table 1 compares the two groups. Of these, 200 were classified as e-money users and 274 as nonusers. For the three actions assessing e-money use, 207 participants reported holding an e-money card or device over a reader, 205 reported adding funds to the e-money balance, and 207 reported understanding the remaining balance. Based on the number of affirmative responses, 262 (55.3%) participants had none, 5 (1.1%) had one, 7 (1.5%) had two, and 200 (42.2%) had all three. Thus, only 12 participants (2.5%) reported one or two actions without meeting the predefined criterion for e-money use.

**Table 1:** Participant characteristics by E-money use status.

Characteristic	Overall *n* = 474^1^	E-money nonusers *n* = 274^1^	E-money users *n* = 200^1^	*P* value
Age, years	73.00 (69.00–77.00)	75.00 (71.00–79.00)	70.50 (67.00–74.50)	<0.001^a^
Sex				0.29^b^
Male	191 (40%)	116 (42%)	75 (38%)	
Female	283 (60%)	158 (58%)	125 (63%)	
Living arrangement				0.59^b^
Living with someone	347 (73%)	198 (72%)	149 (75%)	
Living alone	127 (27%)	76 (28%)	51 (26%)	
Education years, years	12.00 (12.00–14.00)	12.00 (9.00–12.00)	12.00 (12.00–14.00)	<0.001^a^
Physical impairment				0.002^b^
Having physical impairment	101 (21%)	72 (26%)	29 (15%)	
Robust	373 (79%)	202 (74%)	171 (86%)	
Lower cognitive status				0.020^b^
Robust	428 (90%)	240 (88%)	188 (94%)	
Lower cognitive status	46 (9.7%)	34 (12%)	12 (6.0%)	
GDS-15, score	2.00 (1.00–4.00)	2.00 (1.00–4.00)	2.00 (1.00–3.00)	0.011^a^
Current driving habit				<0.001^b^
No	68 (14%)	52 (19%)	16 (8.0%)	
Yes	406 (86%)	222 (81%)	184 (92%)	
General financial management ability				<0.001^b^
Not independent	114 (24%)	88 (32%)	26 (13%)	
Independent	360 (76%)	186 (68%)	174 (87%)	
Employment status				0.062^b^
Paid work	183 (39%)	96 (35%)	87 (44%)	
No paid work	291 (61%)	178 (65%)	113 (57%)	
Marital status				0.72^b^
Married	311 (66%)	180 (66%)	131 (66%)	
Widowed/Divorced	130 (27%)	77 (28%)	53 (27%)	
Never	33 (7.0%)	17 (6.2%)	16 (8.0%)	
Smartphone status				<0.001^c^
Nonownership	62 (13%)	54 (20%)	8 (4.0%)	
Low proficiency	138 (29%)	112 (41%)	26 (13%)	
Moderate proficiency	137 (29%)	74 (27%)	63 (32%)	
High proficiency	137 (29%)	34 (12%)	103 (52%)	

1 n (%); median (Q1–Q3).

^a^ Wilcoxon rank sum test; ^b^Pearson's chi-squared test; ^c^Fisher's exact test.

E-money, electronic money.

The numbers of e-money users by smartphone ownership/proficiency category were 8 of 62 nonowners, 26 of 138 participants with low proficiency, 63 of 137 with moderate proficiency, and 103 of 137 with high proficiency. Compared with nonusers, e-money users were younger, had more years of education, and had lower prevalences of physical impairment and lower cognitive status. Median age was 70.5 years among e-money users and 75.0 years among nonusers. Depressive symptom scores were slightly lower and current driving habit was more common among e-money users. Smartphone ownership/proficiency differed markedly by e-money use. High smartphone proficiency was substantially more common among e-money users, whereas smartphone nonownership and low proficiency were more common among nonusers.

### Association between smartphone ownership/proficiency and E-money use

Modified Poisson regression analyses examined the association between smartphone ownership/proficiency and e-money use, with smartphone nonownership as the reference category. Figure 2-A presents the PRs across the three models. Low smartphone proficiency was not significantly associated with e-money use in any model, whereas moderate and high proficiency were significantly associated with a higher prevalence of e-money use. These associations remained significant after adjustment for social factors, functional factors, and general financial management ability. In the fully adjusted model, participants with moderate smartphone proficiency had a higher prevalence of e-money use than smartphone nonowners (PR = 2.54, 95% CI 1.31–4.93). Participants with high smartphone proficiency also had a higher prevalence of e-money use (PR = 3.65, 95% CI 1.89–7.06). Complete regression results for all participants are presented in Supplementary Table S2.

**Figure 2:**
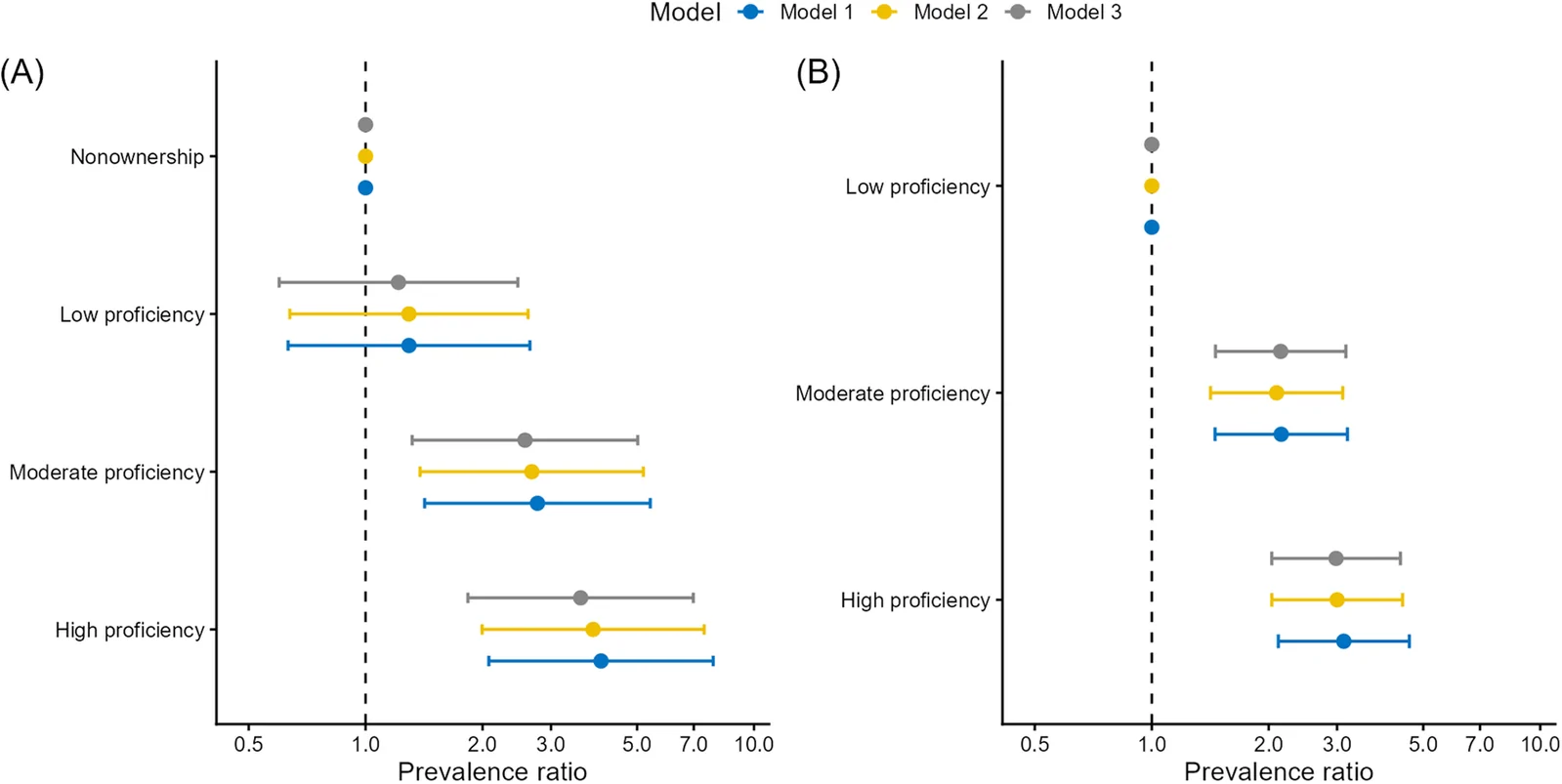
Association between smartphone ownership/proficiency and e-money use. **Panel A** presents the analysis including all participants, with smartphone nonownership as the reference category. **Panel B** presents the analysis restricted to smartphone owners, with low smartphone proficiency as the reference category. Prevalence ratios and 95% confidence intervals were estimated using modified Poisson regression with robust error variance. Model 1 adjusted for age, sex, years of education, living alone, marital status, employment status, and current driving habit. Model 2 additionally adjusted for physical impairment, cognitive impairment, and depressive symptoms. Model 3 further adjusted for general financial management ability. E-money: electronic money.

In the additional analysis restricted to smartphone owners, low smartphone proficiency was the reference category. As shown in Figure 2-B, moderate and high smartphone proficiency were significantly associated with a higher prevalence of e-money use. In the fully adjusted model, the PR was 2.15 (95% CI 1.46–3.16) for moderate proficiency and 2.98 (95% CI 2.04–4.37) for high proficiency compared with low proficiency. Complete regression results for smartphone owners are presented in Supplementary Table S3.

### Sensitivity and robustness analyses

In the sensitivity analysis treating the MDPQ-16 score as a continuous variable among smartphone owners, higher smartphone proficiency was associated with a higher prevalence of e-money use. In the fully adjusted model, each 1 SD increase in the MDPQ-16 score was associated with a PR of 1.44 (95% CI 1.30–1.60), consistent with the main analysis. Results from all three models are presented in Supplementary Table S4.

To assess robustness to potential unmeasured confounding, E-values were calculated for the fully adjusted models. In the main analysis including smartphone nonowners, the E-values for moderate smartphone proficiency were 4.52 for the point estimate and 1.95 for the lower limit of the 95% CI; the corresponding values for high proficiency were 6.66 and 3.15. In the analysis restricted to smartphone owners, the E-values for moderate proficiency were 3.72 for the point estimate and 2.28 for the lower limit of the 95% CI; the corresponding values for high proficiency were 5.41 and 3.50.

## Discussion

This study examined the associations of smartphone ownership and proficiency with e-money use among community-dwelling older adults. The finding that 42.2% of participants were classified as e-money users indicates that e-money is already part of daily financial activities for a substantial proportion of community-dwelling older adults. Moderate and high smartphone proficiency were significantly associated with e-money use even after adjustment for sociodemographic, functional, and financial management-related factors, whereas low proficiency was not. These findings highlight the relevance of practical digital proficiency, in addition to smartphone ownership, to e-money use in later life. Notably, 8 of 62 smartphone nonowners were classified as e-money users, indicating that e-money use does not necessarily require smartphone ownership because card-based payment methods can be used independently. Thus, smartphone ownership and e-money access should not be considered equivalent constructs. This observation further supports examining both smartphone access and proficiency when considering participation in digitally mediated financial activities among older adults.

E-money use may be considered a digitally mediated form of financial IADL. Conventional financial management requires cognitive and practical abilities, including basic monetary skills, financial conceptual knowledge, cash transactions, management of financial records, and financial judgment, all of which are vulnerable to cognitive decline in later life (3, 4, 32). Depending on the service and payment context, e-money use may require additional digitally mediated actions, such as presenting a card or device to a reader, adding funds to the e-money balance, checking the remaining balance, understanding payment procedures, and operating mobile applications or digital devices. These requirements align with the broader concept of digital competence, which encompasses access to digital technologies and skills related to information handling, safety, problem solving, and confident technology use (18, 33). Therefore, e-money use may extend beyond traditional money management by requiring device operation, procedural knowledge, and competence in digital transactions. Collectively, these findings suggest that practical digital proficiency may contribute to e-money use beyond smartphone access.

Importantly, the association between smartphone proficiency and e-money use remained significant after adjustment for cognitive function and general financial management ability. This finding suggests that e-money use cannot be explained solely by conventional financial management capacity, such as the ability to manage money and property independently. Rather, it may reflect a contemporary functional ability integrating financial management and digital device operation (13). This interpretation is consistent with digital financial literacy, which encompasses the knowledge, skills, confidence, and competencies required to safely use digitally delivered financial products and services (11). Recent studies have similarly shown that digital financial literacy reflects skills required for financial transactions in digital environments and is associated with financial well-being (13). Research on financial technology and digital financial inclusion further suggests that digital financial services require financial literacy, awareness of digital products, risk management, and the ability to use digital tools (13). Thus, e-money use among older adults may represent a hybrid activity combining traditional financial management with digital procedural skills.

These findings have practical implications for supporting older adults in increasingly cashless communities. When assessing whether older adults can use e-money, confirming smartphone ownership or access to a payment device alone may be insufficient (34). Assessment should consider abilities required in real-world payment situations, including operating a smartphone or payment application, checking the remaining balance, adding funds, presenting a card or device to a reader, understanding payment procedures, and recognizing basic security risks. The MDPQ may have potential as an initial screening or triage tool to identify older adults who may benefit from more detailed assessment of digital skills and individualized support. Rather than relying on smartphone ownership alone, a brief assessment of practical proficiency may help identify individuals requiring assistance with digitally mediated financial activities. However, because this study was cross-sectional and did not examine individual MDPQ domains in relation to e-money use, the usefulness of the MDPQ as a screening tool and the proficiency domains most relevant to payment tasks require further investigation.

Practical proficiency may represent only one dimension of successful technology use. Previous studies of community-dwelling older adults have suggested that smartphone expertise is also associated with psychological and health-related factors, including anxiety related to technology use (35, 36). Assessment may therefore consider not only whether an older adult can perform specific smartphone or payment-related procedures but also whether fear, anxiety, frustration, or other technology-related concerns interfere with use. Future studies combining smartphone-specific performance measures with measures of technophobia or technology-related anxiety may help identify older adults requiring different types of support. Evidence suggests that interventions to improve digital skills can increase technology use and reduce digital exclusion among older adults, but these interventions should address diverse barriers, including lack of confidence, fear, anxiety, affordability, and accessibility (37). Guidance on digital payments also emphasizes the need for up-to-date knowledge and skills to recognize risks such as fraud, phishing, account hacking, and data theft (38). Support for e-money use should therefore combine practical device training with safety education (37). Health and social care professionals may be well positioned to evaluate e-money use as a daily activity by considering digital skills, task demands, environmental context, and available support. A recent scoping review suggested that occupational therapy interventions using information and communication technology devices may support independence and social connection among community-dwelling older adults while emphasizing individualized support (39). Thus, e-money support should be tailored to individual abilities and living environments rather than assuming that smartphone ownership indicates readiness for digital financial activities.

This study has several limitations. First, its cross-sectional design precludes causal inference; therefore, whether smartphone proficiency promotes e-money use or e-money use improves smartphone proficiency remains unclear.

Second, the e-money assessment did not distinguish among specific electronic payment methods, such as transportation IC cards, credit cards, QR-code payment services, or applications linked to stored credit cards. Because these methods differ in operational procedures and complexity, the three actions assessed by the PADA-D may not apply equally across services. For example, transportation IC cards with an auto-charge function may not require users to actively add funds to the e-money balance. Requiring affirmative responses to all three actions may therefore have classified some regular users as nonusers. Nevertheless, only 12 participants (2.5%) reported one or two actions without meeting the predefined criterion. Future studies should distinguish among payment types and examine service-specific procedural demands and digital proficiency requirements.

Third, because participants volunteered for a community health check-up program, they may have been healthier and more health-conscious than the general older adult population, limiting generalizability.

Fourth, although this study focused on smartphone proficiency, mobile payment adoption among older adults may also be influenced by perceived usefulness, ease of use, trust, perceived risk, and attitudes toward payment technologies (15). These psychological and behavioral factors were not directly assessed.

Fifth, economic resources, including income, wealth, and financial assets, were not directly assessed and represent a plausible source of residual confounding. Previous studies suggest that financial resources may influence cashless payment adoption and use. In a large Japanese study, ePayment adoption was more common among individuals with higher incomes (40), whereas a study of older Korean consumers found that higher income was associated with owning more credit cards and greater monthly credit card expenditure (41). Economic resources may therefore influence opportunities and willingness to engage in cashless financial transactions and partly confound the observed association. For the association between high smartphone proficiency and e-money use, the E-value was 6.66 for the point estimate and 3.15 for the lower confidence limit. Although these E-values do not exclude residual confounding, they indicate that an unmeasured confounder would require relatively strong associations with both smartphone proficiency and e-money use to fully explain the observed association. Future studies should directly assess income, wealth, and other financial resources alongside practical smartphone proficiency and psychological factors related to cashless payment use.

Sixth, smartphone proficiency was assessed only among smartphone owners; therefore, digital proficiency among nonowners was not evaluated. Among smartphone nonowners, 4 participants owned a tablet computer but were classified as nonowners because the exposure was defined specifically by smartphone ownership. Thus, some smartphone nonowners may have had substantial proficiency with other digital devices. Future studies should assess proficiency across multiple device types, including smartphones and tablets, while considering the digital proficiency of individuals who do not currently own these devices.

Despite these limitations, this study has several strengths. We examined actual e-money use among community-dwelling older adults and its association with both smartphone ownership and proficiency while accounting for sociodemographic, physical, cognitive, psychological, and financial management factors. The findings provide insight into e-money use as a contemporary daily activity involving both conventional financial management capacity and practical digital proficiency. Another strength was the recruitment strategy. Participants were not recruited through electronic surveys, online advertisements, or digital links, which may preferentially attract individuals with greater digital access or proficiency. Instead, recruitment relied on pre-distributed response postcards, local publicity campaigns, and newspaper advertisements. This approach may have reduced selection bias related to digital technology use and enabled inclusion of older adults with a broader range of digital experience.

## Conclusion

This study found that, compared with smartphone nonowners, community-dwelling older adults with moderate and high smartphone proficiency had a higher prevalence of e-money use, whereas those with low proficiency showed no statistically significant difference. Among smartphone owners, moderate and high proficiency were also associated with a higher prevalence of e-money use than low proficiency. These findings suggest that e-money use may represent a contemporary form of IADL reflecting not only conventional financial management capacity but also the ability to adapt to digitally mediated environments. Support for older adults in increasingly cashless communities should therefore address not only smartphone or payment device ownership but also practical proficiency in using these technologies in daily life.

## Data Availability

The raw data supporting the conclusions of this article will be made available by the authors, without undue reservation.
