# Supplementary material for: Association between smartphone ownership, proficiency, and electronic money use among community-dwelling older adults: a cross-sectional study
Source: Front Digit Health. 2026 Oct 20;8:1947033. doi: 10.3389/fdgth.2026.1947033 (PMC13727751; doi:10.3389/fdgth.2026.1947033)
Supplement: Supplementary file 1 [file Table1.docx]

**Table S1. STROBE Checklist for Reporting the Present Cross-Sectional Study**

|  | Item No | Recommendation | Page |
| --- | --- | --- | --- |
| **Title and abstract** | 1 | (*a*) Indicate the study’s design with a commonly used term in the title or the abstract | P1,L1-2; P3,L7-8 |
|  |  | (*b*) Provide in the abstract an informative and balanced summary of what was done and what was found | P3,L2-30 |
| Introduction | | |  |
| Background/rationale | 2 | Explain the scientific background and rationale for the investigation being reported | P4,L2-30; P5,L1-29; P6,L1-6 |
| Objectives | 3 | State specific objectives, including any prespecified hypotheses | P6,L7-15 |
| Methods | | |  |
| Study design | 4 | Present key elements of study design early in the paper | P6,L20-30 |
| Setting | 5 | Describe the setting, locations, and relevant dates, including periods of recruitment, exposure, follow-up, and data collection | P6,L20-23 |
| Participants | 6 | (*a*) Give the eligibility criteria, and the sources and methods of selection of participants | P6,L20-28; Figure 1 |
| Variables | 7 | Clearly define all outcomes, exposures, predictors, potential confounders, and effect modifiers. Give diagnostic criteria, if applicable | P7,L13-27; P8,L1-31; P9,L1-31; P10,L4-32; P11,L1-5 |
| Data sources/ measurement | 8* | For each variable of interest, give sources of data and details of methods of assessment (measurement). Describe comparability of assessment methods if there is more than one group | P7,L13-27; P8,L1-31; P9,L9-31; P10,L4-32; P11,L1-5 |
| Bias | 9 | Describe any efforts to address potential sources of bias | P9,L27-P10,L1; P11,L15-31; P12,L6-18; P21,L21-26 |
| Study size | 10 | Explain how the study size was arrived at | P6,L20-28; Figure 1 |
| Quantitative variables | 11 | Explain how quantitative variables were handled in the analyses. If applicable, describe which groupings were chosen and why | P9,L1-6; P9,L11-15; P10,L7-27 |
| Statistical methods | 12 | (*a*) Describe all statistical methods, including those used to control for confounding | P11,L8-31; P12,L1-25 |
|  |  | (*b*) Describe any methods used to examine subgroups and interactions | P12,L1-5 |
|  |  | (*c*) Explain how missing data were addressed | P6,L24-27 |
|  |  | (*d*) If applicable, describe analytical methods taking account of sampling strategy | N/A |
|  |  | (*e*) Describe any sensitivity analyses | P12,L6-18 |
| Results | | |  |
| Participants | 13* | (a) Report numbers of individuals at each stage of study—eg numbers potentially eligible, examined for eligibility, confirmed eligible, included in the study, completing follow-up, and analysed | P6,L20-28; Figure 1 |
|  |  | (b) Give reasons for non-participation at each stage | P6,L24-27; Figure 1 |
|  |  | (c) Consider use of a flow diagram | P6,L27-28; P7, Figure 1 |
| Descriptive data | 14* | (a) Give characteristics of study participants (eg demographic, clinical, social) and information on exposures and potential confounders | P13,L7-16; P13,L19-P15,L8; Table 1 |
|  |  | (b) Indicate number of participants with missing data for each variable of interest | P6,L24-27 |
| Outcome data | 15* | Report numbers of outcome events or summary measures | P13,L1-9 |
| Main results | 16 | (*a*) Give unadjusted estimates and, if applicable, confounder-adjusted estimates and their precision (eg, 95% confidence interval). Make clear which confounders were adjusted for and why they were included | P11,L25-31; P15,L11-30; Supplementary Tables S2-S3 |
|  |  | (*b*) Report category boundaries when continuous variables were categorized | P9,L1-6; P10,L7-22 |
|  |  | (*c*) If relevant, consider translating estimates of relative risk into absolute risk for a meaningful time period | N/A |
| Other analyses | 17 | Report other analyses done—eg analyses of subgroups and interactions, and sensitivity analyses | P15,L24-30; P16,L13-P17,L5; Supplementary Tables S3-S4 |
| Discussion | | |  |
| Key results | 18 | Summarise key results with reference to study objectives | P17,L8-21 |
| Limitations | 19 | Discuss limitations of the study, taking into account sources of potential bias or imprecision. Discuss both direction and magnitude of any potential bias | P19,L29-P21,L14 |
| Interpretation | 20 | Give a cautious overall interpretation of results considering objectives, limitations, multiplicity of analyses, results from similar studies, and other relevant evidence | P17,L8-P19,L28; P20,L1-P21,L14; P21,L29-P22,L8 |
| Generalisability | 21 | Discuss the generalisability (external validity) of the study results | P20,L14-16; P21,L7-14; P21,L21-26 |
| Other information | | |  |
| Funding | 22 | Give the source of funding and the role of the funders for the present study and, if applicable, for the original study on which the present article is based | P23,L20-24 |
