# Supplementary material for: Association between smartphone ownership, proficiency, and electronic money use among community-dwelling older adults: a cross-sectional study
Source: Front Digit Health. 2026 Oct 20;8:1947033. doi: 10.3389/fdgth.2026.1947033 (PMC13727751; doi:10.3389/fdgth.2026.1947033)
Supplement: Supplementary file 2 [file Table2.docx]

**Table S2. Full results of modified Poisson regression analyses for e-money use among all participants**

| **Variable** | **Model 1, PR (95% CI)** | **Model 1, P value** | **Model 2, PR (95% CI)** | **Model 2, P value** | **Model 3, PR (95% CI)** | **Model 3, P value** |
| --- | --- | --- | --- | --- | --- | --- |
| Low smartphone proficiency (ref: nonownership) | 1.29 (0.63–2.65) | 0.482 | 1.29 (0.64–2.62) | 0.475 | 1.22 (0.60–2.47) | 0.589 |
| Moderate smartphone proficiency (ref: nonownership) | 2.77 (1.42–5.41) | 0.003 | 2.68 (1.38–5.19) | 0.004 | 2.54 (1.31–4.93) | 0.006 |
| High smartphone proficiency (ref: nonownership) | 4.04 (2.08–7.85) | <.001 | 3.85 (1.99–7.44) | <.001 | 3.65 (1.89–7.06) | <.001 |
| Age, years | 0.97 (0.94–0.99) | 0.004 | 0.96 (0.94–0.99) | 0.003 | 0.97 (0.94–0.99) | 0.006 |
| Male (ref: female) | 1.28 (1.05–1.56) | 0.013 | 1.28 (1.06–1.56) | 0.011 | 1.15 (0.95–1.39) | 0.157 |
| Education, years | 1.06 (1.01–1.10) | 0.016 | 1.05 (1.01–1.10) | 0.027 | 1.05 (1.00–1.10) | 0.042 |
| Living alone (ref: living with someone) | 0.91 (0.66–1.27) | 0.584 | 0.92 (0.67–1.27) | 0.616 | 0.92 (0.67–1.26) | 0.590 |
| Widowed/Divorced (ref: married) | 1.39 (1.00–1.94) | 0.050 | 1.38 (1.00–1.90) | 0.050 | 1.32 (0.96–1.81) | 0.086 |
| Never married (ref: married) | 1.15 (0.81–1.64) | 0.426 | 1.16 (0.81–1.66) | 0.420 | 1.08 (0.77–1.53) | 0.650 |
| Not engaged in remunerative work (ref: yes) | 1.06 (0.88–1.28) | 0.522 | 1.09 (0.90–1.30) | 0.381 | 1.05 (0.88–1.26) | 0.597 |
| Current driving habit: Yes (ref: no) | 1.51 (0.98–2.34) | 0.063 | 1.45 (0.95–2.22) | 0.088 | 1.47 (0.97–2.22) | 0.071 |
| No physical impairment (ref: physical impairment) | - |  | 1.23 (0.92–1.66) | 0.164 | 1.26 (0.94–1.70) | 0.122 |
| Lower cognitive status (ref: robust) | - |  | 0.80 (0.51–1.25) | 0.333 | 0.80 (0.51–1.27) | 0.344 |
| GDS-15, score | - |  | 0.97 (0.93–1.01) | 0.168 | 0.98 (0.94–1.03) | 0.422 |
| Independent in general financial management (ref: not independent) | - |  | - |  | 1.68 (1.22–2.31) | 0.002 |
| Note. The numbers of e-money users/total participants were 8/62 for smartphone nonownership, 26/138 for low proficiency, 63/137 for moderate proficiency, and 103/137 for high proficiency. Model 1 was adjusted for age, sex, years of education, living alone, marital status, employment status, and current driving habit. Model 2 was additionally adjusted for physical impairment, cognitive status, and depressive symptoms. Model 3 was further adjusted for general financial management ability. PR, prevalence ratio; CI, confidence interval; GDS-15, 15-item Geriatric Depression Scale. | | | | | | |
