# Supplementary material for: Association between smartphone ownership, proficiency, and electronic money use among community-dwelling older adults: a cross-sectional study
Source: Front Digit Health. 2026 Oct 20;8:1947033. doi: 10.3389/fdgth.2026.1947033 (PMC13727751; doi:10.3389/fdgth.2026.1947033)
Supplement: Supplementary file 3 [file Table3.docx]

**Table S3. Full results of modified Poisson regression analyses for e-money use among smartphone owners**

| **Variable** | **Model 1, PR (95% CI)** | **Model 1, P value** | **Model 2, PR (95% CI)** | **Model 2, P value** | **Model 3, PR (95% CI)** | **Model 3, P value** |
| --- | --- | --- | --- | --- | --- | --- |
| Moderate smartphone proficiency (ref: low proficiency) | 2.15 (1.46–3.19) | <.001 | 2.10 (1.42–3.10) | <.001 | 2.15 (1.46–3.16) | <.001 |
| High smartphone proficiency (ref: low proficiency) | 3.12 (2.12–4.60) | <.001 | 3.00 (2.04–4.42) | <.001 | 2.98 (2.04–4.37) | <.001 |
| Age, years | 0.97 (0.95–0.99) | 0.012 | 0.97 (0.95–0.99) | 0.009 | 0.97 (0.95–1.00) | 0.019 |
| Male (ref: female) | 1.26 (1.04–1.53) | 0.019 | 1.26 (1.04–1.53) | 0.019 | 1.14 (0.94–1.38) | 0.183 |
| Education, years | 1.06 (1.02–1.11) | 0.006 | 1.06 (1.01–1.11) | 0.011 | 1.06 (1.01–1.10) | 0.017 |
| Living alone (ref: living with someone) | 0.92 (0.66–1.28) | 0.632 | 0.93 (0.67–1.29) | 0.658 | 0.93 (0.68–1.27) | 0.628 |
| Widowed/Divorced (ref: married) | 1.33 (0.96–1.86) | 0.091 | 1.32 (0.95–1.83) | 0.093 | 1.26 (0.92–1.73) | 0.145 |
| Never married (ref: married) | 1.19 (0.84–1.69) | 0.334 | 1.18 (0.83–1.67) | 0.362 | 1.10 (0.79–1.54) | 0.565 |
| Not engaged in remunerative work (ref: yes) | 1.04 (0.86–1.25) | 0.678 | 1.06 (0.88–1.27) | 0.524 | 1.03 (0.86–1.23) | 0.782 |
| Current driving habit: Yes (ref: no) | 1.51 (0.97–2.35) | 0.070 | 1.45 (0.94–2.23) | 0.095 | 1.46 (0.96–2.22) | 0.074 |
| No physical impairment (ref: physical impairment) | - |  | 1.26 (0.94–1.70) | 0.124 | 1.30 (0.97–1.75) | 0.084 |
| Lower cognitive status (ref: robust) | - |  | 0.74 (0.46–1.18) | 0.205 | 0.74 (0.45–1.20) | 0.219 |
| GDS-15, score | - |  | 0.98 (0.94–1.03) | 0.388 | 0.99 (0.95–1.04) | 0.767 |
| Independent in general financial management (ref: not independent) | - |  | - |  | 1.63 (1.19–2.24) | 0.002 |
| Note. Among smartphone owners, the numbers of e-money users/total participants were 26/138 for low proficiency, 63/137 for moderate proficiency, and 103/137 for high proficiency. The analysis was restricted to smartphone owners. Low smartphone proficiency was used as the reference category. Model 1 was adjusted for age, sex, years of education, living alone, marital status, employment status, and current driving habit. Model 2 was additionally adjusted for physical impairment, cognitive status, and depressive symptoms. Model 3 was further adjusted for general financial management ability. PR, prevalence ratio; CI, confidence interval; GDS-15, 15-item Geriatric Depression Scale. | | | | | | |
