# Supplementary material for: Association between smartphone ownership, proficiency, and electronic money use among community-dwelling older adults: a cross-sectional study
Source: Front Digit Health. 2026 Oct 20;8:1947033. doi: 10.3389/fdgth.2026.1947033 (PMC13727751; doi:10.3389/fdgth.2026.1947033)
Supplement: Supplementary file 4 [file Table4.docx]

**Table S4. Sensitivity Analysis Treating the MDPQ-16 Score as a Continuous Variable Among Smartphone Owners**

| Variable | Model 1  PR  CI | Model 1  P value | Model 2  PR  CI | Model 2  P value | Model 3  PR  CI | Model 3  P value |
| --- | --- | --- | --- | --- | --- | --- |
| MDPQ-16 score (per 1SD increase) | 1.48 (1.34–1.64) | <.001 | 1.45 (1.31–1.62) | <.001 | 1.44 (1.30–1.60) | <.001 |
| Age, years | 0.97 (0.95–0.99) | 0.012 | 0.97 (0.95–0.99) | 0.009 | 0.97 (0.95–1.00) | 0.018 |
| Male (ref: female) | 1.32 (1.08–1.61) | 0.007 | 1.31 (1.07–1.61) | 0.008 | 1.21 (0.99–1.47) | 0.068 |
| Education, years | 1.06 (1.01–1.11) | 0.009 | 1.06 (1.01–1.11) | 0.016 | 1.05 (1.01–1.10) | 0.025 |
| Living alone (ref: living with someone) | 0.94 (0.69–1.28) | 0.692 | 0.94 (0.69–1.29) | 0.705 | 0.94 (0.69–1.27) | 0.676 |
| Widowed/divorced (ref: married) | 1.33 (0.96–1.83) | 0.082 | 1.31 (0.96–1.80) | 0.092 | 1.26 (0.93–1.71) | 0.134 |
| Never married (ref: married) | 1.13 (0.80–1.60) | 0.489 | 1.12 (0.79–1.58) | 0.516 | 1.06 (0.76–1.47) | 0.745 |
| Not engaged in remunerative work (ref: yes) | 1.03 (0.86–1.24) | 0.719 | 1.05 (0.88–1.27) | 0.579 | 1.02 (0.85–1.23) | 0.796 |
| Current driving habit: Yes (ref: no) | 1.52 (0.98–2.34) | 0.061 | 1.45 (0.95–2.23) | 0.085 | 1.46 (0.97–2.21) | 0.069 |
| No physical impairment (ref: physical impairment) | - |  | 1.25 (0.93–1.68) | 0.137 | 1.28 (0.96–1.72) | 0.097 |
| Lower cognitive status (ref: robust) | - |  | 0.75 (0.46–1.22) | 0.242 | 0.75 (0.46–1.23) | 0.253 |
| GDS-15, score | - |  | 0.98 (0.94–1.03) | 0.433 | 0.99 (0.95–1.04) | 0.778 |
| Independent in general financial management (ref: not independent) | - |  | - |  | 1.57 (1.14–2.17) | 0.006 |
| Note. Model 1 was adjusted for age, sex, years of education, living alone, marital status, employment status, and current driving habit. Model 2 was additionally adjusted for physical impairment, cognitive status, and depressive symptoms. Model 3 was further adjusted for general financial management ability. PR, prevalence ratio; CI, confidence interval; MDPQ-16, Mobile Device Proficiency Questionnaire short version; SD, standard deviation; GDS-15, 15-item Geriatric Depression Scale. | | | | | | |
